# Polymorphisms of C242T and A640G in CYBA Gene and the Risk of Coronary Artery Disease: A Meta-Analysis

**DOI:** 10.1371/journal.pone.0084251

**Published:** 2014-01-02

**Authors:** Qiumei Xu, Fangfen Yuan, Xuemei Shen, Hui Wen, Wei Li, Bei Cheng, Jing Wu

**Affiliations:** 1 Key Laboratory of Environment and Health, Ministry of Education & Department of Epidemiology and Biostatistics, School of Public Health, Tongji Medical College, Huazhong University of Science and Technology, Wuhan, China; 2 Department of Gerontology, Union Hospital, Tongji Medical College, Huazhong University of Science and Technology, Wuhan, China; Children’s National Medical Center, Washington, United States of America

## Abstract

**Background:**

Coronary artery disease (CAD) is a leading cause of mortality in many countries. Considerable studies have been carried out to investigate the relationship between the C242T and A640G polymorphisms of CYBA gene and CAD, but the results were still inconsistent. Hence we conducted a meta-analysis to clarify the association.

**Methods and Results:**

A total of 21 eligible literatures were included in the meta-analysis. We observed a significant decreased risk of CAD for C242T polymorphism in Asian population under an allelic model (OR 0.75; 95% CI 0.67–0.84) and a dominant model (OR 0.69; 95% CI 0.61–0.79), however, in overall population and other population no significant association was revealed. We also found A640G polymorphism may contribute to reducing CAD risk under an allelic model (OR 0.84; 95% CI 0.75–0.93), dominant model (OR0.77; 95% CI 0.64–0.92) and recessive model (OR0.82; 95% CI 0.69–0.97). No publication bias was found.

**Conclusion:**

Our meta-analysis confirmed a protective effect of C242Tpolymorphism on CAD in Asian population and indicated that A640G polymorphism was significantly associated with decreased risk of CAD.

## Introduction

Coronary artery disease (CAD), also namely ischemic heart disease (IHD) or coronary heart disease (CHD), mainly including angina and myocardial infarction (MI), is still a leading cause of mortality in many countries with three-fourths of global deaths due to CAD in the low- and middle-income countries [1]. According to a report issued by the World Health Organization, the CAD death toll was expected to account for 13.4% of the total population death by the year 2030 [2]. The death caused by CAD in men and women aged ≥60 years, is surpassed only by human immunodeficiency virus/acquired immune deficiency syndrome in persons aged 15 to 59 years [3]. Oxidative stress in the vasculature and even the whole body induced by superoxide anion via the NADH/NADPH pathway has been implicated in atherosclerosis which is the basic and initial pathogenesis of CAD [4]–[6]. Besides, evidence has also shown that oxidative stress contributes to the development of cardiovascular diseases by vascular wall remodeling and endothelial dysfunction [7].

Evidence over recent years has indicated that the predominant cellular source of superoxide anion in the context of cardiovascular diseases is the NADPH oxidase family [8], [9], which is a class of membrane-associated enzymes that catalyzes the one electron reduction of oxygen to produce reactive oxygen species (ROS) using NADH or NADPH as the electron donor [10]. Among the components of NADPH oxidase, the p22phox protein, an essential subunit for the activation of the NADPH oxidase [11], is expressed in various cells such as human endothelial cells and vascular smooth muscle cells [12]. In addition, the higher expression of p22phox was found in human atherosclerotic coronary arteries than in non atherosclerotic arteries [13].

P22-phox is encoded by the CYBA gene, which is located on chromosome 16q24 and consists of six exons and five introns [14] with a length of 8.5 kb. A large number of genetic variations of this gene, such as C242T, A640G,-930A/G, -675A/T and C549T polymorphism, have been reported. Among these polymorphisms, the two polymorphisms of C242T and A640G have been extensively studied and considered the most interesting. The C242T polymorphism is located in exon 4 and results in an amino acid substitution (histidine to tyrosine) [14], which leads to a loss of oxidative function and a decreased production of ROS and oxidative stress in the vasculature [15]. An *in-vivo* study [16] also showed the C242T allele was associated with reduced NAD(P)H oxidase activity in human blood vessels. While the A640G polymorphism is located in 3′ untranslated region (3′UTR) [17] of CYBA gene with no amino acid substitution and has also been found an effect on the ROS generation [18], [19]. It has been assumed that A640G modified the stability of mRNA and translational activity of CYBA through the interaction with other regions of mRNA [18].

Since Inoue first found T allele of the C242T polymorphism might have a protective effect against CAD [20], the association of the CYBA gene C242T polymorphism with CAD has been extensively studied over the last decade, however, the results have been conflicting. Nasti even stated the opposite effect with T allele as a risk factor for CAD [21]. As to the association of CYBA gene A640G polymorphism with CAD, the related studies are relatively fewer, but controversy still exists. For example, Inoue found no association between A640G polymorphism and CAD [20], whereas, Gardemann’s study suggested a protective effect on CAD exerted by the G allele [22]. So we conducted this meta-analysis integrating previous publications to study the association between the two polymorphisms of CYBA gene and CAD.

## Materials and Methods

### Search Strategy and Identification of Relevant Studies

An extensive literature searching of PubMed, EMBASE, ISI Web of Science and Chinese Wan Fang Data was performed for relevant articles without restricting language from June 1996 to May 2012, using the combinations of the keywords “Coronary artery disease”, “Coronary heart disease”, “ischemic heart disease”, “angina pectoris”, “myocardial infarction”, “Polymorphism”, “CYBA”, “P22phox”, “C242T”, “A640G”, “NADPH oxidase”. References of reviews and retrieved studies were also scanned and request to the author to access to the paper has been also tried. We conducted the meta-analysis and reported its results according to the Preferred Reporting Items for Systematic Reviews and Meta-Analysis (PRISMA) statement (Checklist S1).

The following inclusion criteria had to be fulfilled: (1) case-control study or large-scale replication study assessing the association between C242T polymorphism, A640G polymorphism and CAD risk as an original study. (2) Numbers of case and control groups or available data for calculating genotypic OR with 95% CI. (3)The CAD cases defined as coronary heart disease (CHD), coronary artery disease (CAD), myocardial infarction (MI), acute myocardial infarction (AMI), or unstable angina (UA).

### Data Extraction

All the data were extracted independently by two reviewers who reached a consensus on all of the items. Following information was extracted from the eligible literature: first author’s last name, year of publication, country, ethnic origin of the studied population, definition of cases, number in case and control groups, genotype distributions, male percentage and mean ages in case and control groups.

### Statistical Analysis

Pooled effect was calculated for the allelic model, dominant model and recessive model in both C242T polymorphism and A640G polymorphism respectively.

Heterogeneity among studies was assessed using *Q* test [23] and Higgins *I* ^2^ [24], and was considered significant when *P*<0.05 for Q statistic. Then heterogeneity was qualified by *I^2^*: *I^2^* = 0%–30%, no or marginal between-study heterogeneity; *I^2^* = 30%–75%, mild heterogeneity; *I^2^* = 75%–100%, notable heterogeneity [25]. A fixed effect model (Mantel-Haenszel method) was applied when there is no heterogeneity (*P*<0.05), otherwise a random effect model (DerSimonian and Laird method) was adopted [26]. A meta-regression model was employed to explore the sources of the heterogeneity [27] and then we carried out stratified analysis by subgroup. Sensitivity analysis was conducted to assess the influence of each study on overall pooled OR, with sequential omission of individual study [28]. Funnel plot and Egger’s test described by Egger et al [29] for funnel plot asymmetry were applied to evaluate the evidence for publication bias. All statistical analyses were carried out with *R* software (version 15.3) and a probability value of *P*<0.05 was considered statistically significant.

## Results

### Characteristics of Included Studies

**Figure 1:**
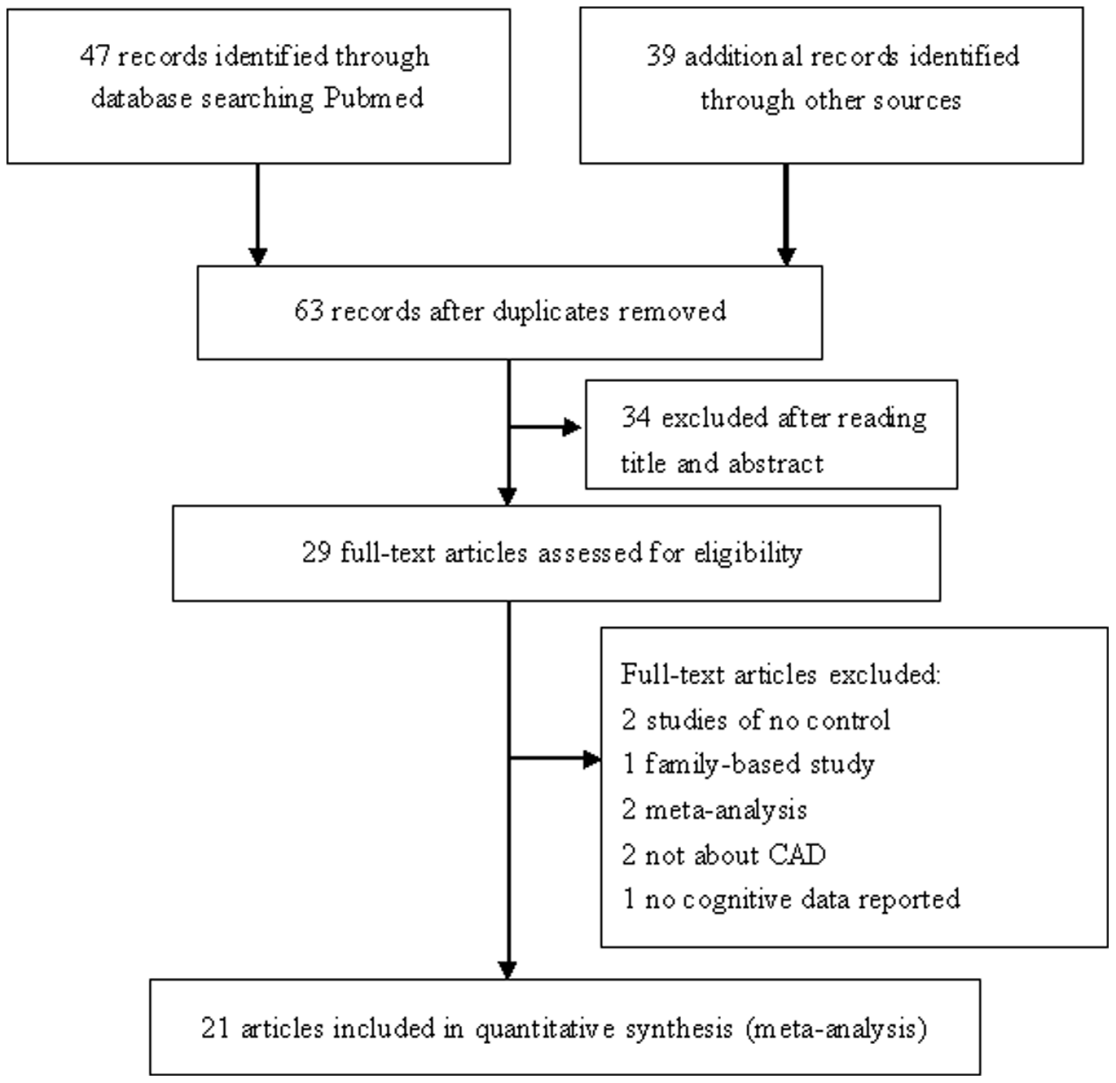
Flow chart of the literature search. CAD, coronary artery disease.

The study selection process is shown in Figure 1. A comprehensive search identified 86 references. After removing the duplicate literature and reports, a total of 21 publications [20]–[22], [30]–[47] preliminarily fit the inclusion criteria. However, after further examination, we removed Morgan’s study that was a large-scale application study with 1461 participants and 85 genetic variants indicating that none of the variants was unequivocally validated. Since 1 article included two populations, both of them were considered as an independent study. There were totally 21studies included in the meta-analysis. The characteristics of these studies were listed in Table 1. There were 7 studies based on Asian population, 10 studies conducted in Caucasian population and 4 studies from other population, such as Spanish, American. The diagnoses in the included articles were CHD, UA, MI, AMI and CAD. For C242T polymorphism, 20 studies were available with a total of 8845 cases and 6855 controls, while for A640G polymorphism; only 6 studies covered a total of 2399 cases and 1411 controls.

**Table 1:** Basic characteristics of the included studies.

Study	Year	Country	Ethnicity	Disease	Genotype							Sample size	% of male (case/control)	Age mean (case/control)
					Case				Control					
C242T					TT	TC	CC	TT		TC	CC			
Inoue	1998	Japan	Asian	CAD	2	26	173	0		53	148	201/201	0.70/0.70	59.8/54.9
Gardemann	1999	German	Caucasian	CAD	207	748	751	65		208	226	1706/499	1.00/1.00	62.2/61.4
Li	1999	America	Other	CAD	27	70	52	14		42	47	149/103	0.77/0.48	60/57
Cai	1999	Australia	Caucasian	CAD	65	250	235	13		62	64	550/139	0.80/0.47	56.9/54.5
Saha	1999	India	Asian	CHD	17	66	43	20		77	57	126/154	0.88/0.93	55.2/53.9
Saha	1999	China	Asian	CHD	3	23	125	3		25	139	151/167	1.00/1.00	55.3/54.5
Lee	2001	Korean	Asian	CAD	4	40	261	1		49	165	305/215	1.00/1.00	56.1/50.2
Stanger	2001	Australia	Caucasian	CAD	15	50	43	6		24	15	108/45	1.00/1.00	52.9/54.2
Yamada	2002	Japan	Asian	MI	25	319	1440	16		242	816	1784/1074	1.00/1.00	62.1/62.0
Zafari	2002	America	Other	CAD	21	70	58	6		26	18	164/52	0.99/0.94	61.7/57.1
Balague	2004	Spain	Caucasian	AMI	2	41	61	2		47	57	104/106	0.88/0/89	50.2/50.4
Murase	2004	Japan	Asian	MI/UA	2	67	384	5		170	587	593/1595	0.76/0.48	50.0/60.6
Fan	2006	Finland	Caucasian	CAD	12	66	172	10		56	86	250/152	0.77/0.58	62.0/55.0
Nasti	2006	Italy	Caucasian	CAD	37	147	92	25		95	98	276/218	0.83/0.68	65.5/66.2
He MA	2007	China	Asian	CAD/MI	2	32	531	2		64	543	565/609	0.57/0.52	61.9/64.7
Niemiec	2007	Poland	Caucasian	CAD	25	80	67	18		85	66	172/169	0.67/0.70	43.8/34.2
Reyes	2008	Spain	Other	MI/UA	48	137	119	47		145	123	304/315	0.78/0.74	56.0/54.5
Alexey	2010	Russia	Caucasian	CAD	34	91	188	4		41	87	313/132	0.54/0.57	59.2/55.5
Goliasch	2011	Austria	Caucasian	AMI	11	47	41	31		79	82	99/192	0.87/0.90	37.3/34.7
Najafi	2012	Italy	Caucasian	CAD	23	47	44	12		32	24	114/68	0.68/0.32	62.8/55.9
**A640G**					**GG**	**AG**	**AA**	**GG**		**AG**	**AA**			
Inoue	1998	Japan	Asian	CAD	82	83	36	80		79	42	201/201	0.70/0.70	59.8/54.9
Gardemann	1999	German	Caucasian	CAD	313	735	438	141		251	107	1486/499	1.00/1.00	62.2/61.4
Zafari	2002	America	Other	CAD	37	75	37	13		25	12	149/50	0.99/0.94	61.7/57.1
Reyes	2008	Spain	Other	MI/UA	104	155	46	48		165	96	305/309	0.78/0.74	56.0/54.5
Goliasch	2011	Austria	Caucasian	AMI	23	47	28	39		103	50	98/192	0.87/0.90	37.3/34.7
Niemiec	2011	Poland	Caucasian	CAD	39	68	53	40		75	45	160/160	0.68/0.76	43.8/59.3

Abbreviations: N, Number of studies OR, Odds ratio; 95% CI, 95% confidence interval; REM, random-effects model; FEM, fix-effects model.

### Results of the Overall Meta-analysis

**Figure 2:**
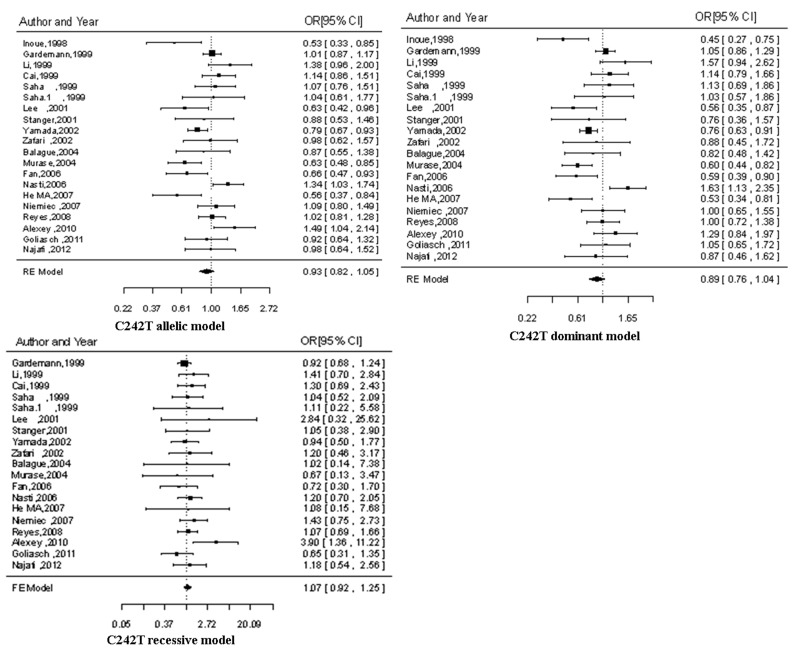
The forest plots of OR with 95% CI for C242T polymorphism and CAD risk. OR, Odds ratio; 95% CI, 95% confidence interval; RE, random-effects model; FE, fix-effects model; CAD, coronary artery disease.

**Figure 3:**
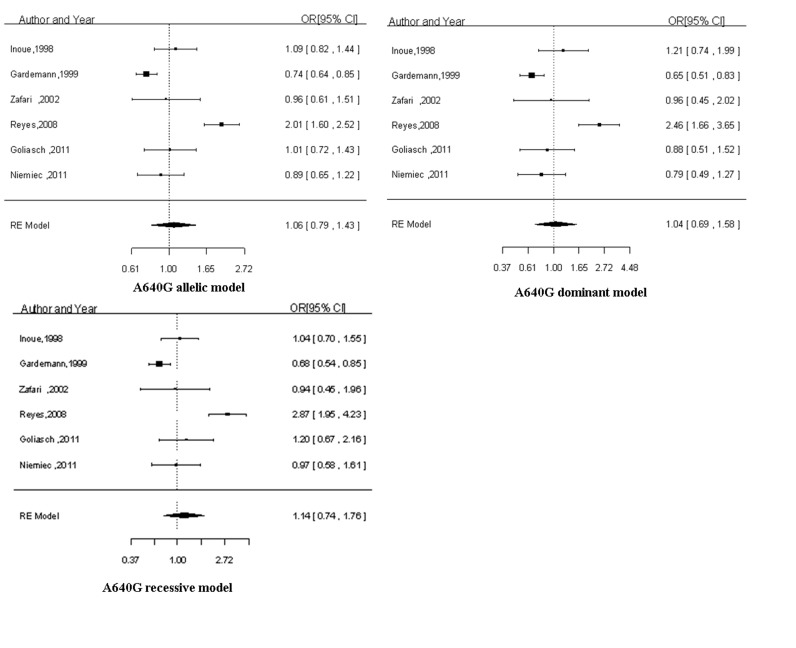
The forest plots of OR with 95% CI for A640G polymorphism and CAD risk. OR, Odds ratio; 95% CI, 95% confidence interval; RE, random-effects model; FE, fix-effects model; CAD, coronary artery disease.

Figure 2 and Figure 3 summarizes the ORs with corresponding 95% CIs for the association between C242T and A640G polymorphisms in the p22phox gene and the risk for CAD in the allelic, dominant and recessive models. A random-effect model was applied to the allelic model and dominant model in the study of C242T polymorphism and to all the genetic models in the study of A640G polymorphism, while a fixed-effect model was chosen for the recessive model in C242T polymorphism according to the *P* values for heterogeneity. For both C242T polymorphism and A640G polymorphism, no significant association was observed under all the three genetic models. The results are presented in Table 2.

**Table 2:** Initial pooled estimates and stratified analysis for the association between polymorphisms and CAD.

Genetic Model	N	Model for analysis	OR (95% CI)	*P* for heterogeneity	*I* ^2^ (%)	*P* for Egger’s test
C242T						
Allelic model	20	REM	0.93(0.82,1.05)	<0.0001	67.88	0.6768
**Asian**	**7**	**FEM**	**0.75(0.67,0.84)**	**0.0618**	**52.16**	**0.6371**
Caucasian	10	FEM	1.04(0.95,1.14)	0.0599	48.31	0.7246
Other	3	FEM	1.09(0.91,1.30)	0.3400	10.75	0.7679
Dominant model	20	REM	0.89(0.76,1.04)	<0.0001	68.15	0.9351
**Asian**	**7**	**FEM**	**0.69(0.61,0.79)**	**0.0522**	**52.30**	**0.7497**
Caucasian	10	FEM	1.04(0.93,1.18)	0.0685	47.86	0.4538
Other	3	FEM	1.09(0.85,1.40)	0.2730	21.63	0.8526
Recessive model	19	FEM	1.07(0.92,1.25)	0.8181	0.00	0.2440
A640G						
Allele contrast	6	REM	1.06(0.79,1.43)	<0.0001	86.73	0.5087
Dominant model	6	REM	1.04(0.69,1.58)	<0.0001	80.75	0.4531
Recessive model	6	REM	1.14(0.74,1.76)	<0.0001	83.45	0.4144

Abbreviations: N, Number of studies OR, Odds ratio; 95% CI, 95% confidence interval; REM, random-effects model; FEM, fix-effects model.

### Meta-regression Analysis and Stratified Analysis

To explore the source of heterogeneity, a meta-regression analysis of C242T under the allelic model and the dominant model respectively was performed. We conducted a series univariate model by adding single covariates including ethnicity, publication year, disease, sex and age. In the univariate analysis, only the ethnicity can explain the between-study heterogeneity in allelic model (*P* = 0.0154, *tau^2^* reducing from 0.0506 to 0.0335) and dominant model (*P* = 0.0140, *tau^2^* reducing from 0.0807 to 0.0518).The meta-regression results were presented at Table S1. After stratified by ethnicity, heterogeneity in the subgroup decreased and a decreased CAD risk was conferred in Asian population both in the allelic model (OR = 0.75, 95% CI = 0.67–0.84) and the dominant model (OR = 0.69, 95% CI = 0.61–0.79). Nonetheless, in Caucasian population and other population, no significant association between C242T and CAD was still found (Table 2).

### Sensitivity Analysis

The results for C242T polymorphism showed that none of the studies dramatically affected the combined results under the allelic model and the recessive model, while under the dominant model the overall result changed significantly after removing Nasti’s study [21] with OR(95% CI) changing from 0.89(0.76–1.04) to 0.85(0.73–0.99), though the heterogeneity did not decrease sharply (*I^2^* = 62.53%) (Table S2).This may be because of the marginal statistical significance and moderate effect of the polymorphism. In the sensitivity analysis on A640G polymorphism, in view of the considerable heterogeneity caused by Reyes’s study [43] which probably resulted from the population admixture in the study, we removed it and found a significant association between A640G and CAD under an allelic model (OR 0.84; 95% CI 0.75–0.93), dominant model (OR 0.77; 95% CI 0.64–0.92) and recessive model (OR 0.82;95% CI 0.69–0.97) ) and no statistically significant heterogeneity existed (Table S2). We will have a more detailed discussion in the following part. The adjusted results were showed in Table 3.

**Table 3:** Adjusted pooled measure on the relation of A640G polymorphism to CAD after sensitivity analysis.

Genetic model	N	Model for analysis	OR (95% CI)	*P* for heterogeneity	*I* ^2^(%)	*P* for Egger’s test
A640G						
**Allele contrast**	**5**	**FEM**	**0.84(0.75,0.93)**	**0.0923**	**49.08**	**0.0741**
**Dominant model**	**5**	**FEM**	**0.77(0.64,0.92)**	**0.2346**	**35.92**	**0.1073**
**Recessive model**	**5**	**FEM**	**0.82(0.69,0.97)**	**0.1851**	**40.69**	**0.0755**

Abbreviations: N, Number of studies OR, Odds ratio; 95% CI, 95% confidence interval; FEM, fix-effects model.

### Publication Bias

**Figure 4:**
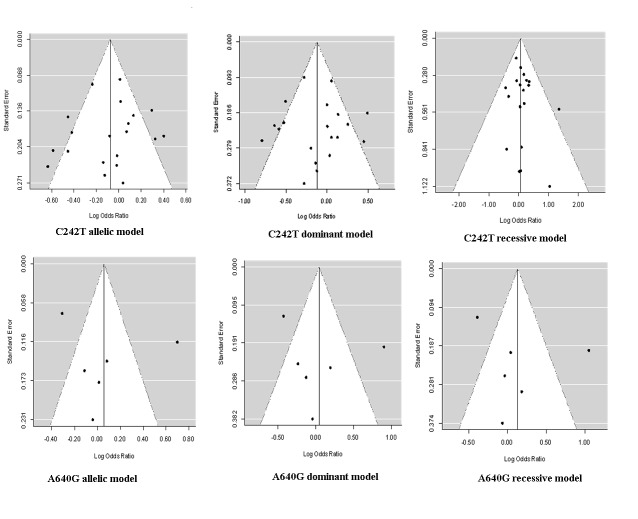
The funnel plots of natural logarithm of OR against inverse standard error in each study.

As demonstrated by the funnel plot and the Egger’s test, there was no significant publication bias in any overall meta-analysis with all *P* for Egger’s test>0.05 (Table 2, Figure 4).

## Discussion

Due to the key role of p22phox in CAD and the conflicting results about the relationship of its polymorphisms and CAD, this meta-analysis was conducted and the following conclusions were drawn: (1) The C242T polymorphism of CYBA gene provided significantly protective effect on CAD in Asian populations under an allelic model (OR 0.75; 95% CI 0.67–0.84) and a dominant model (OR 0.69; 95% CI 0.61–0.79), while no protective effect was found in Caucasian population and other population, just consistent with the results in Di Castelnuovo A, et al’s meta-analysis [48] which provided great reference for our study. (2) A significant association between p22phox gene A640G polymorphism and CAD was found under an allelic model (OR 0.84; 95% CI 0.75–0.93), dominant model (OR 0.77; 95% CI 0.64–0.92) and recessive model (OR 0.82; 95% CI 0.69–0.97).

In a Japanese population Inoue [20] firstly found 242T as a decreased risk of CAD. Subsequently, Lee [35] and He [40] confirmed that T variant allele was significantly associated with reduced CAD risk both in Korean and Chinese populations. However, Zafari [31] and Cai [33] negated this conclusion in American and Australian populations respectively. Our meta-analysis, of 19 articles with 20 populations from different ethnic origin containing 8845 cases and 6855 controls, afforded us a much higher possibility to reach the reasonable conclusions, which were just consistent with the results in Fang’s meta-analysis [49]. The T allele had contributed to the reduced NADH-stimulated superoxide production and decreased NAD(P)H oxidase reactivity, with reduced activity (by about 30%) of the phagocytic enzyme after stimulation with phorbol 12-myristate 13-acetate (PMA) [16], [50], indicating a weak protective role, which may account for the Asian population as suggested by our findings. Such conflicting results in Asian populations and non-Asian populations are likely to exist because multiple long-standing risk factors confound the possible effect of this polymorphism on CAD such as diabetes, smoke, hypertension and hypercholesterolaemia. Nevertheless, caution is needed in conclusion drawing due to our limited subgroup samples and attention should be paid to the heterogeneity, which still existed though decreased after a subgroup analysis because of the relatively large meta-analysis and the inevitable between-study heterogeneity.

For the first time this meta-analysis was conducted to explore the association between p22phox gene A640G polymorphism and CAD. And interestingly, we found a significant association between A640G and CAD under three genetic models after removing Reyes’s study [43]. In view of the considerable heterogeneity caused by this study and to obtain a stable result, we removed this study for reanalysis which hardly affected the power of our meta-analysis (the sample size dropped from 2399 cases and 1411 controls to 2094 cases and 1102 controls) and thus, the final results can be acceptable. In a functional study, Wyche et al. [50] indeed did not confirm any changes in superoxide production by the A640G polymorphism. However, Gardemann et al. [22] found that the A640G SNP might modify processing or stability of p22phox mRNA or alternatively act as a neutral marker. Schirmer et al. [19] observed a significantly reduced ROS formation with increasing numbers of 640G variant alleles in a study of the functional significance of A640G, suggesting a protective effect of the A640G polymorphism on CAD. All these evidence supported a protective effect of 640G on CAD. Nevertheless, notation should be paid to the results because of the limited samples.

In conclusion, this meta-analysis was a renewed and confirmed study to assess the association between C242T polymorphism and CAD. It’s the first time that a meta-analysis was conducted to summarize the relationship of A640G polymorphism and CAD. Our combined results implicated the p22phox gene C242T and A640G SNPs played a vital role in coronary artery disease, collectively confirming a genetic involvement of the two polymorphisms in CAD. Further large and well-designed studies will be needed to clarify the association of the polymorphisms and CAD risk. Additional meta-analysis based on GWAS data will also be essential in the future.

## Supporting Information

Table S1 The meta-regression analysis for heterogeneity under the allelic model and dominant model of p22phox gene C242T polymorphism.
(DOC)

Table S2 Sensitive analysis of pooled OR.
(DOCX)

Checklist S1 PRISMA 2009 Checklist.
(DOCX)

## References

[1] Gaziano TA, Bitton A, Anand S, Abrahams-Gessel S, Murphy A, et al (2010) Growing epidemic of coronary heart disease in low- and middle-income countries. Current Problems in Cardiology 35: 72–115. 20109979 10.1016/j.cpcardiol.2009.10.002 PMC2864143

[2] Mathers CD, Loncar D (2006) Projections of global mortality and burden of disease from 2002 to 2030. PLoS Medicine 3: e442. 17132052 10.1371/journal.pmed.0030442 PMC1664601

[3] McKay J, Mensah GA, Greenlund K, Mendis S (2004) The atlas of heart disease and stroke. World Health Organization. 405 p.

[4] San JG, Fortuno A, Beloqui O, Diez J, Zalba G( 2008) NADPH oxidase CYBA polymorphisms, oxidative stress and cardiovascular diseases. Clinical Science (London) 114: 173–182. 10.1042/CS20070130 18184111

[5] Epstein FH, Diaz MN, Frei B, Vita JA, Keaney JF (1997) Antioxidants and atherosclerotic heart disease. New England Journal of Medicine 337: 408–416. 9241131 10.1056/NEJM199708073370607

[6] Singh U, Jialal I (2006) Oxidative stress and atherosclerosis. Pathophysiology 13: 129–142. 16757157 10.1016/j.pathophys.2006.05.002

[7] Fortuno A, José GS, Moreno MU, Díez J, Zalba G (2005) Oxidative stress and vascular remodelling. Experimental Physiology 90: 457–462. 15890797 10.1113/expphysiol.2005.030098

[8] Mueller CF, Laude K, McNally JS, Harrison DG (2005) Redox mechanisms in blood vessels. Arteriosclerosis, Thrombosis, and Vascular Biology 25 274–278. 10.1161/01.ATV.0000149143.04821.eb 15514203

[9] Mohazzab K, Kaminski PM, Wolin MS (1994) NADH oxidoreductase is a major source of superoxide anion in bovine coronary artery endothelium. American Journal of Physiology-Heart and Circulatory Physiology 266: H2568–H2572. 10.1152/ajpheart.1994.266.6.H2568 8024019

[10] Griendling KK, Sorescu D, Ushio-Fukai M (2000) NAD (P) H oxidase role in cardiovascular biology and disease. Circulation Research 86: 494–501. 10720409 10.1161/01.res.86.5.494

[11] Sumimoto H, Hata K, Mizuki K, Ito T, Kage Y, et al (1996) Assembly and activation of the phagocyte NADPH oxidase specific interaction of the N-terminal Src homology 3 domain of p47phox with p22phox is required for activation of the NADPH oxidase. Journal of Biological Chemistry 271: 22152–22158. 8703027 10.1074/jbc.271.36.22152

[12] Ushio-Fukai M, Zafari AM, Fukui T, Ishizaka N, Griendling KK (1996) P22phox is a critical component of the superoxide-generating NADH/NADPH oxidase system and regulates angiotensin iiinduced hypertrophy in vascular smooth muscle cells. Journal of Biological Chemistry 271: 23317–23321. 8798532 10.1074/jbc.271.38.23317

[13] Azumi H, Inoue N, Takeshita S, Rikitake Y, Kawashima S, et al (1999) Expression of NADH/NADPH oxidase p22phox in human coronary arteries. Circulation 100: 1494–1498. 10510050 10.1161/01.cir.100.14.1494

[14] Dinauer MC, Pierce EA, Bruns GA, Curnutte JT, Orkin SH (1990) Human neutrophil cytochrome b light chain (p22-phox). Gene structure, chromosomal location, and mutations in cytochrome-negative autosomal recessive chronic granulomatous disease. Journal of Clinical Investigation 86: 1729–1737. 2243141 10.1172/JCI114898 PMC296926

[15] Whitehead AS, FitzGerald GA (2001) Twenty-first century phox: not yet ready for widespread screening. Circulation 103: 7–9. 11136676 10.1161/01.cir.103.1.7

[16] Guzik TJ, West N, Black EJ, McDonald E, Ratnatunga D (2000) Functional effect of the C242T polymorphism in the NAD(P)H oxidase p22phox gene on vascular superoxide production in atherosclerosis. Circulation 102: 1744–1747. 11023926 10.1161/01.cir.102.15.1744

[17] de Boer M, de Klein A, Hossle JP, Seger R, Corbeel L, et al. (1992) Cytochrome b558-negative, autosomal recessive chronic granulomatous disease: two new mutations in the cytochrome b558 light chain of the NADPH oxidase (p22-phox). American Journal of Human Genetics 51: p. 1127–1135. PMC1682833 1415254

[18] Bedard K, Attar H, Bonnefont J, Jaquet V, Borel C, et al (2009) Three common polymorphisms in the CYBA gene form a haplotype associated with decreased ROS generation. Human Mutation 30: 1123–1133. 19388116 10.1002/humu.21029

[19] Schirmer M, Hoffmann M, Kaya E, Tzvetkov M, Brockmöller J (2007) Genetic polymorphisms of NAD(P)H oxidase: variation in subunit expression and enzyme activity. The Pharmacogenomics Journal 8: 297–304. 17684477 10.1038/sj.tpj.6500467

[20] Inoue N, Kawashima S, Kanazawa K, Yamada S, Akita H, et al (1998) Polymorphism of the NADH/NADPH oxidase p22 phox gene in patients with coronary artery disease. Circulation 97: 135–137. 9445163 10.1161/01.cir.97.2.135

[21] Nasti S, Spallarossa P, Altieri P, Garibaldi S, Fabbi P, et al (2006) C242T Polymorphism in CYBA gene (p22 phox and risk of coronary artery disease in a population of Caucasian Italians. Disease Markers 22: 167–173. 16788250 10.1155/2006/458587 PMC3851654

[22] Gardemann A, Mages P, Katz N, Tillmanns H, Haberbosch W (1999) The p22 phox A640G gene polymorphism but not the C242T gene variation is associated with coronary heart disease in younger individuals. Atherosclerosis 145: 315–323. 10488959 10.1016/s0021-9150(99)00083-0

[23] Lau J, Loannidis JP, Schmid CH (1997) Quantitative synthesis in systematic reviews. Annals of Internal Medicine 127: 820–826. 9382404 10.7326/0003-4819-127-9-199711010-00008

[24] Higgins JP, Thompson SG, Deeks JJ, Altman DG (2003) Measuring inconsistency in meta-analyses. British Medical Journal 327: 557–560. 12958120 10.1136/bmj.327.7414.557 PMC192859

[25] Higgins J, Thompson SG (2002) Quantifying heterogeneity in a meta-analysis. Statistics in Medicine 21: 1539–1558. 12111919 10.1002/sim.1186

[26] Hedges LV, Vevea JL (1998) Fixed-and random-effects models in meta-analysis. Psychological Methods 3: 486–504.

[27] Thompson SG, Higgins J (2002) How should meta-regression analyses be undertaken and interpreted? Statistics in Medicine 21: 1559–1573. 12111920 10.1002/sim.1187

[28] Patsopoulos NA, Evangelou E, Loannidis JP (2008) Sensitivity of between-study heterogeneity in meta-analysis: proposed metrics and empirical evaluation. International Journal of Epidemiology 37: 1148–1157. 18424475 10.1093/ije/dyn065 PMC6281381

[29] Egger M, Davey Smith G, Schneider M, Minder C (1997) Bias in meta-analysis detected by a simple, graphical test. British Medical Journal 315: 629–634. 9310563 10.1136/bmj.315.7109.629 PMC2127453

[30] Stanger O, Renner W, Khoschsorur G, Rigler B, Wascher TC (2001) NADH/NADPH oxidase p22 phox C242T polymorphism and lipid peroxidation in coronary artery disease. Clinical Physiology 21: 718–722. 11722480 10.1046/j.1365-2281.2001.00381.x

[31] Zafari AM, Davidoff MN, Austin H, Valppu L, Cotsonis G, et al (2002) The A640G and C242T p22 phox polymorphisms in patients with coronary artery disease. Antioxidants and Redox Signaling 4: 675–680. 12230880 10.1089/15230860260220184

[32] Saha N, Sanghera D, Kamboh M (1999) The p22 phox polymorphism C242T is not associated with CHD risk in Asian Indians and Chinese. European Journal of Clinical investigation 29: 999–1002. 10583446 10.1046/j.1365-2362.1999.00575.x

[33] Cai H, Duarte N, Wilcken DEL, Wang XL (1999) NADH/NADPH oxidase p22 phax C242T polymorphism and coronary artery disease in the Australian population. European Journal of Clinical Investigation 29: 744–748. 10469162 10.1046/j.1365-2362.1999.00531.x

[34] Li A, Prasad A, Mincemoyer R, Satorius C, Epstein N, et al (1999) Relationship of the C242T p22phox gene polymorphism to angiographic coronary artery disease and endothelial function. American Journal of Medical Genetics 86: 57–61. 10440830 10.1002/(sici)1096-8628(19990903)86:1<57::aid-ajmg11>3.0.co;2-r

[35] Lee WH, Hwang TH, Oh GT, Kwon S, UkCho YH, et al (2001) Genetic factors associated with endothelial dysfunction affect the early onset of coronary artery disease in Korean males. Vascular Medicine 6: 103–108. 11530961

[36] Yamada Y, Izawa H, Ichihara S, Ishihara H, Hirayama H, et al (2002) Prediction of the risk of myocardial infarction from polymorphisms in candidate genes. New England Journal of Medicine 347: 1916–1923. 12477941 10.1056/NEJMoa021445

[37] Mata-Balaguer T, de la Herrán R, Ruiz-Rejón C, Ruiz-Rejón M, Garrido-Ramos MA, et al (2004) Angiotensin-converting enzyme and p22 (phox) polymorphisms and the risk of coronary heart disease in a low-risk Spanish population. International Journal of Cardiology 95: 145–151. 15193812 10.1016/j.ijcard.2003.05.017

[38] Murase Y, Yamada Y, Hirashiki A, Ichihara S, Kanda H, et al (2004) Genetic risk and gene-environment interaction in coronary artery spasm in Japanese men and women. European Heart Journal 25: 970–977. 15172469 10.1016/j.ehj.2004.02.020

[39] Fan M, Kahonen M, Rontu R, Lehtinen R, Viik J, et al (2006) The p22phox C242T gene polymorphism is associated with a reduced risk of angiographically verified coronary artery disease in a high-risk Finnish Caucasian population. The Finnish Cardiovascular Study. American Heart Journal 152: 538–42. 16923427 10.1016/j.ahj.2006.02.018

[40] He MA, Cheng LX, Jiang CZ, Zeng HS, Wang J, et al (2007) Associations of polymorphism of P22(phox) C242T, plasma levels of vitamin E, and smoking with coronary heart disease in China. American Heart Journal 153 : 640. e1–640: e6. 10.1016/j.ahj.2007.01.002 17383305

[41] Morgan TM, Krumholz HM, Lifton RP, Spertus JA (2007) Nonvalidation of reported genetic risk factors for acute coronary syndrome in a large-scale replication study. the Journal of the American Medical Association 297: 1551–1561. 17426274 10.1001/jama.297.14.1551

[42] Niemiec P, Zak I, Wita K (2007) The 242T variant of the CYBA gene polymorphism increases the risk of coronary artery disease associated with cigarette smoking and hypercholesterolemia. Coronary Artery Disease 18: 339–346. 17627182 10.1097/MCA.0b013e328241d97a

[43] Macias-Reyes A, Rodriguez-Esparragon F, Caballero-Hidalgo A, Hernandez-Trujillo Y, Medina A, et al (2008) Insight into the role of CYBA A640G and C242T gene variants and coronary heart disease risk. A case-control study. Free Radical Research 42: 82–92. 18324526 10.1080/10715760701796918

[44] Nikitin AG, Chistiakov DA, Minushkina LO, Zateyshchikov DA, Nosikov VV, et al (2010) Association of the CYBA, PPARGC1A, PPARG3, and PPARD gene variants with coronary artery disease and metabolic risk factors of coronary atherosclerosis in a Russian population. Heart and Vessels 25: 229–236. 20512451 10.1007/s00380-009-1159-9

[45] Goliasch G, Wiesbauer F, Grafl A, Ponweiser E, Blessberger H, et al (2011) The effect of p22-PHOX (CYBA) polymorphisms on premature coronary artery disease (≤40 years of age). Thrombosis and Haemostasis 105: 529–534. 21136016 10.1160/TH10-08-0529

[46] Najafi M, Alipoor B, Shabani M, Amirfarhangi A, Ghasemi H, et al (2012) Association between rs4673 (C/T) and rs13306294 (A/G) haplotypes of NAD (P) H oxidase p22phox gene and severity of stenosis in coronary arteries. Gene 499: 213–217. 22410402 10.1016/j.gene.2012.02.032

[47] Niemiec P, Nowak T, Balcerzyk A, Krauze J, Zak I (2011) The CYBA gene A640G polymorphism influences predispositions to coronary artery disease through interactions with cigarette smoking and hypercholesterolemia. Biomarkers 16: 405–412. 21777168 10.3109/1354750X.2011.580368

[48] Di Castelnuovo A, Soccio M, Iacoviello L, Evangelista V, Consoli A, et al (2008) The C242T polymorphism of the p22phox component of NAD(P)H oxidase and vascular risk. Two case-control studies and a meta-analysis. Thromb Haemost 99: 594–601. 18327409 10.1160/TH07-08-0480

[49] Fang S, Wang L, Jia C (2010) Association of p22phox gene C242T polymorphism with coronary artery disease: A meta-analysis. Thrombosis Research 125: e197–e201. 20100625 10.1016/j.thromres.2010.01.001

[50] Wyche KE, Wang SS, Griendling KK, Dikalov SI, Austin H, et al (2004) C242T CYBA polymorphism of the NADPH oxidase is associated with reduced respiratory burst in human neutrophils. Hypertension 43: 1246–1251. 15078863 10.1161/01.HYP.0000126579.50711.62

