# Supplementary material for: Polymorphisms of C242T and A640G in CYBA Gene and the Risk of Coronary Artery Disease: A Meta-Analysis
Source: PLoS One. 2014 Jan 2;9(1):e84251. doi: 10.1371/journal.pone.0084251 (PMC3879292; doi:10.1371/journal.pone.0084251)
Supplement: Table S1 — The meta-regression analysis for heterogeneity under the allelic model and dominant model of p22phox gene C242T polymorphism. (DOC) [file pone.0084251.s001.doc]

**Table S1.**The meta-regression analysis for heterogeneity under the allelic model and dominant model of p22phox gene C242T polymorphism.

| Variable | Coefficient | *P* value | *Tau*^2^ value |
| --- | --- | --- | --- |
| Allelic model |  |  |  |
| **ethnicity** | **0.1345** | **0.0154** | **0.0335** |
| year | 0.0060 | 0.6967 | 0.0548 |
| disease | -0.0395 | 0.3410 | 0.0498 |
| sex | -0.0004 | 0.9744 | 0.0554 |
| age | 0.0033 | 0.7683 | 0.0550 |
| Dominant model |  |  |  |
| **ethnicity** | **0.1725** | **0.0140** | **0.0518** |
| year | 0.0057 | 0.7712 | 0.0870 |
| disease | -0.0414 | 0.4320 | 0.0817 |
| sex | 0.0061 | 0.7050 | 0.0867 |
| age | 0.0049 | 0.7316 | 0.0866 |
