# Supplementary material for: Polymorphisms of C242T and A640G in CYBA Gene and the Risk of Coronary Artery Disease: A Meta-Analysis
Source: PLoS One. 2014 Jan 2;9(1):e84251. doi: 10.1371/journal.pone.0084251 (PMC3879292; doi:10.1371/journal.pone.0084251)
Supplement: Table S2 — Sensitive analysis of pooled OR. (DOCX) [file pone.0084251.s002.docx]

**Table S2.**Sensitive analysis of pooled OR**.**

| Study omitted | OR (95% CI) | *P* for heterogeneity | *I^2^*(%) | Study omitted | OR (95% CI) | *P* for heterogeneity | *I^2^*(%) |
| --- | --- | --- | --- | --- | --- | --- | --- |
| **C242T** |  |  |  |  |  |  |  |
| **Allelic model** |  |  |  | **Recessive model** |  |  |  |
| Inoue | 0.95(0.84, 1.07) | 0.0002 | 65.27 | Gardemann | 1.13(0.94 ,1.36) | 0.8463 | 0.00 |
| Gardemann | 0.92(0.80,1.05) | <0.0001 | 66.91 | Li | 1.06(0.90, 1.24) | 0.8043 | 0.00 |
| Li | 0.91(0.80, 1.03) | 0.0001 | 66.55 | Cai | 1.06(0.90, 1.24) | 0.7907 | 0.00 |
| Cai | 0.91(0.80, 1.04) | <0.0001 | 68.58 | Saha | 1.07(0.91,1.26) | 0.7667 | 0.00 |
| Saha | 0.92(0.80,1.05) | <0.0001 | 69.58 | Saha | 1.07(0.92,1.25) | 0.7664 | 0.00 |
| Saha | 0.92(0.81,1.05) | <0.0001 | 69.86 | Lee | 1.07(0.91,1.25) | 0.8133 | 0.00 |
| Lee | 0.94(0.83,1.07) | 0.0001 | 67.25 | Stanger | 1.07(0.91,1.26) | 0.7665 | 0.00 |
| Stanger | 0.93(0.81,1.06) | <0.0001 | 69.94 | Yamada | 1.08(0.92,1.27) | 0.7776 | 0.00 |
| Yamada | 0.94(0.82,1.07) | 0.0001 | 66.51 | Zafari | 1.07(0.91,1.25) | 0.7699 | 0.00 |
| Zafari | 0.92(0.81,1.05) | <0.0001 | 70.00 | Balague | 1.07(0.92,1.25) | 0.7665 | 0.00 |
| Balague | 0.93(0.81,1.06) | <0.0001 | 69.96 | Murase | 1.08(0.92,1.26) | 0.7862 | 0.00 |
| Murase | 0.95(0.84,1.07) | 0.0003 | 64.37 | Fan | 1.09(0/93,1.27) | 0.8193 | 0.00 |
| Fan | 0.94(0.83,1.07) | 0.0001 | 66.83 | Nasti | 1.06(0.90,1.25) | 0.7772 | 0.00 |
| Nasti | 0.91(0.80,1.03) | 0.0003 | 64.50 | He MA | 1.07(0.92,1.25) | 0.7663 | 0.00 |
| He MA | 0.95(0.84,1.07) | 0.0002 | 64.67 | Niemiec | 1.05(0.90,1.24) | 0.8155 | 0.00 |
| Niemiec | 0.92(0.80,1.05) | <0.0001 | 69.32 | Reyes | 1.07(0.91,1.27) | 0.7664 | 0.00 |
| Reyes | 0.92(0.80,1.05) | <0.0001 | 69.09 | Alexey | 1.04(0.89,1.22) | 0.9874 | 0.00 |
| Alexey | 0.90(0.80,1.02) | 0.0002 | 64.50 | Goliasch | 1..10(0.93,1.25) | 0.8732 | 0.00 |
| Goliasch | 0.93(0.81,1.06) | <0.0001 | 70.05 | Najafi | 1.07(0.91,1.25) | 0.7703 | 0.00 |
| Najafi | 0.92(0.81,1.05) | <0.0001 | 70.02 | **A640G** |  |  |  |
| **Dominant model** |  |  |  | **Allele contrast** |  |  |  |
| Inoue | 0.91(0.78, 1.07) | 0.0002 | 66.61 | Inoue | 1.06(0.74,1.51) | <0.0001 | 89.18 |
| Gardemann | 0.87(0.74 ,1.03) | 0.0001 | 69.29 | Gardemann | 1.16(0.85,1.58) | 0.0001 | 79.93 |
| Li | 0.86(0.74, 1.01) | 0.0001 | 68.29 | Zafari | 1.08(0.76,1.52) | <0.0001 | 90.01 |
| Cai | 0.87(0.74, 1.03) | <0.0001 | 71.31 | **Reyes** | **0.84(0.75,0.93)** | **0.0923** | **49.08** |
| Saha | 0.87(0.74,1.03) | <0.0001 | 71.76 | Goliasch | 1.07(0.75,1.53) | <0.0001 | 89.73 |
| Saha | 0.88(0.75,1.04) | <0.0001 | 68.93 | Niemiec | 1.10(0.77,1.56) | <0.0001 | 88.98 |
| Lee | 0.91(0.77,1.06) | 0.0001 | 71.68 | **Dominantmodel** |  |  |  |
| Stanger | 0.89(0.76,1.05) | <0.0001 | 68.90 | Inoue | 1.01(0.62,1.66) | <0.0001 | 84.23 |
| Yamada | 0.90(0.76,1.06) | 0.0001 | 71.86 | Gardemann | 1.18(0.76,1.83) | 0.0020 | 72.60 |
| Zafari | 0.89(0.75,1.04) | <0.0001 | 71.94 | Zafari | 1.06(0.66, 1.70) | <0.0001 | 85.32 |
| Balague | 0.90(0.78,1.05) | <0.0001 | 67.96 | **Reyes** | **0.77(0.64,0.92)** | **0.2346** | **35.92** |
| Murase | 0.89(0.76,1.04) | 0.0002 | 68.15 | Goliasch | 1.08(0.66,1.76) | <0.0001 | 84.52 |
| Fan(2006) | 0.90(0.78,1.05) | 0.0001 | 69.40 | Niemiec | 1.10(0.68, 1.79) | <0.0001 | 83.26 |
| **Nasti** | **0.85(0.73,0.99)** | **0.0011** | **62.53** | **Recessive model** |  |  |  |
| He MA | 0.91(0.78,1.07) | 0.0002 | 67.70 | Inoue | 1.17(0.69,1.96) | <0.0001 | 85.71 |
| Niemiec | 0.88(0.74,1.04) | <0.0001 | 71.81 | Gardemann | 1.31(0.84,2.04) | 0.0009 | 74.86 |
| Reyes | 0.88(0.74,1.04) | <0.0001 | 71.51 | Zafari | 1.18(0.72,1.93) | <0.0001 | 87.33 |
| Alexey | 0.87(0.74,1.02) | 0.0001 | 69.84 | **Reyes** | **0.82(0.69,0.97)** | **0.1851** | **40.69** |
| Goliasch | 0.88(0.74,1.04) | <0.0001 | 71.66 | Goliasch | 1.13(0.68,1.89) | <0.0001 | 87.28 |
| Najafi | 0.89(0.75,1.05) | <0.0001 | 71.91 | Niemiec | 1.18(0.71,1.97) | <0.0001 | 86.67 |
